# AAV-Mediated Expression of Human VEGF, TNF-α, and IL-6 Induces Retinal Pathology in Mice

**DOI:** 10.1167/tvst.10.11.15

**Published:** 2021-09-14

**Authors:** Carina M. Weigelt, Holger Fuchs, Tanja Schönberger, Birgit Stierstorfer, Benjamin Strobel, Thorsten Lamla, Thomas Ciossek, Remko A. Bakker, Norbert H. Redemann

**Affiliations:** 1 Cardiometabolic Diseases Research, Boehringer Ingelheim Pharma GmbH & Co. KG, Biberach an der Riß, Germany; 2 Drug Discovery Sciences, Boehringer Ingelheim Pharma GmbH & Co. KG, Biberach an der Riß, Germany; 3 Research Beyond Borders, Boehringer Ingelheim Pharma GmbH & Co. KG, Biberach an der Riß, Germany

**Keywords:** retina, adeno-associated virus, mouse, retinopathy, gene transfer

## Abstract

**Purpose:**

Retinopathies display complex pathologies, including vasculopathies, inflammation, and fibrosis, leading ultimately to visual impairment. However, animal models accurately reflecting these pathologies are lacking. In this study, we evaluate the suitability of using Adeno-associated virus (AAV)-mediated long-term expression of cytokines to establish retinal pathology in the murine retina.

**Methods:**

We administered recombinant, Müller-glia targeted AAV-ShH10 into the mouse vitreous to induce retinal expression of either human vascular endothelial growth factor (VEGF)-A_165_, tumor necrosis factor alpha (TNF-α), or interleukin-6 (IL-6) and evaluated consequent effects by optical coherence tomography, fluorescein angiography, and histology.

**Results:**

Intravitreal injection of AAVs resulted in rapid and stable expression of the transgenes within 1 to 6 weeks. Akin to the role of VEGF-A in wet age-related macular degeneration, expression of VEGF-A led to several vasculopathies in mice, including neovascularization and vascular leakage. In contrast, the expression of the proinflammatory cytokines TNF-α or IL-6 induced retinal inflammation, as indicated by microglial activation. Furthermore, the expression of TNF-α, but not of IL-6, induced immune cell infiltration into the vitreous as well as vasculitis, and subsequently induced the development of fibrosis and epiretinal membranes.

**Conclusions:**

In summary, the long-term expression of human VEGF-A_165_, TNF-α, or IL-6 in the mouse eye induced specific pathologies within 6 weeks that mimic different aspects of human retinopathies.

**Translational Relevance:**

AAV-mediated expression of human genes in mice is an attractive approach to provide valuable insights into the underlying molecular mechanisms causing retinopathies and is easily adaptable to other genes and preclinical species supporting drug discovery for retinal diseases.

## Introduction

Retinopathies, such as age-related macular degeneration (AMD) or diabetic retinopathy (DR), display a complex disease etiology and cause progressive visual impairment. Underlying pathologies include vascular dysfunction, inflammation, and fibrosis. Animal models resembling pathway-specific parts of these human pathologies are essential for studying and understanding the underlying molecular mechanisms. Frequently used animal models for retinopathies include both genetic models as well as acute, transient models, such as laser photocoagulation,^1^ and may not fully reflect the progressive development of the human disease.

Adeno-associated virus (AAV) vectors are a well-known tool for gene transfer in vitro and in vivo and allow efficient, long-term expression of a gene of interest. A variety of capsids and promoters for AAVs has been introduced, allowing cell-type specific expression of transgenes. For example, AAVs with the well-characterized ShH10 capsid primarily infect Müller glia in the murine retina.^2^ AAVs can be readily introduced into the eye by intravitreal (IVT) or subretinal injection to transduce cells in the retina and are a valuable approach for both mechanistic preclinical studies as well as for clinical gene therapy approaches.^3^ Here, we use recombinant AAVs to induce the expression of three retinopathy-associated human genes to study their function in vivo in murine retinae.

Vascular endothelial growth factor (VEGF)-A_165_ is a well-known growth factor that is upregulated in many retinopathies, including wet-AMD,^4^ DR,^5^^,^^6^ diabetic macular edema (DME),^7^ retinopathy of prematurity (ROP),^8^ and neovascular glaucoma,^9^ as well as in in vitro models of oxidative stress in retinal pigment epithelium (RPE) cells.^10^ Anti-VEGF-A antibody treatments reduce vascular leakage and improve vision in patients with wet-AMD^11^ and DME.^12^ To study the function of VEGF-A and develop treatments, VEGF-A-induced animal models have been established. IVT injection of recombinant VEGF-A has been shown to induce acute vascular leakage and/or neovascularization in rats,^13^ rabbits,^14^ and primates.^15^ However, the effects are transient due to the short half-live of injected proteins in the vitreous. To avoid this limitation, several transgenic mouse models expressing VEGF-A in the mouse retina have been generated.^16^^–^^19^ Depending on the obtained expression levels, these VEGF-A transgenic mice develop a range of phenotypes, including vascular leakage,^16^^,^^17^^,^^19^^,^^20^ neovascularization,^16^^,^^19^ and retinal detachment.^16^^,^^18^ In contrast to transgenic models, AAVs can be readily generated and allow for greater flexibility regarding the desired expression levels and localization, as well as the preclinical species of choice. For example, subretinal injection of AAVs to induce retinal VEGF-A^21^^–^^24^ expression has been shown to result in vascular leakage and/or neovascularization, in rodents as well as primates, demonstrating per se that AAV-mediated expression is a feasible approach to study effects of VEGF-A in vivo.

Tumor necrosis factor alpha (TNF-α) is a well-known proinflammatory cytokine that mediates the recruitment and activation of monocytes.^25^ TNF-α is upregulated in different retinopathies, such as proliferative DR^26^ and uveitis.^27^ Treatment of uveitis with anti-TNF-α antibodies reduces ocular inflammation.^28^ In vivo studies have demonstrated that IVT injection of recombinant TNF-α activates microglia and leads to optic nerve degeneration and loss of retinal ganglion cells (RGCs) in mice and rats.^29^^,^^30^ Transgenic mice overexpressing human TNF-α have been generated and used for arthritis studies,^31^ however, to the best of our knowledge, effects on the retina have not been described. Recently, Da Cunha et al. demonstrated that AAV-mediated expression of mouse TNF-α leads to immune cell infiltration into the vitreous, but a detailed characterization of this model is still missing.^32^

Interleukin-6 (IL-6) is another proinflammatory cytokine induced in retinal diseases, such as DR,^6^^,^^33^ DME,^7^^,^^34^ proliferative vitreoretinopathy (PVR),^35^ and uveitis.^36^ IL-6 signaling is also regulated in in vitro models of oxidative stress induced by A2E in RPE cells.^37^ IVT injection of recombinant IL-6 has been performed in several studies with variable outcomes. In some studies, IL-6 induced an uveitis-like phenotype^38^ or infiltration of macrophages into the subretinal space.^39^ In contrast, other studies have not observed ocular inflammation upon either IVT administration of recombinant IL-6^40^^,^^41^ or AAV-mediated expression of mouse IL-6.^32^ Although IL-6 knock-out mice have been used to study the function of IL-6 in retinopathies and found that loss-of IL-6 may be beneficial in mouse models of glaucoma^42^ and PVR,^43^ exacerbated photoreceptor loss was observed in a retinal detachment model.^44^ Altogether, the role of IL-6 is controversial, suggesting a bi-functional role for IL-6 that may be beneficial or detrimental depending on the context as well as the model used.

Herein, we used AAVs to express human VEGF-A_165_, TNF-α, and IL-6 in the murine eye. The IVT injection of AAV-VEGF induced vascular pathologies, including vascular leakage and retinal neovascularization. The expression of TNF-α induced strong inflammation in the mouse retinae, including immune cell infiltration into the vitreous, as well as retinal vasculitis and fibrosis. In contrast, expression of IL-6 induced immune cell activation and infiltration into the subretinal space. Altogether, AAVs expressing three different retinopathy-associated genes led to phenotypes resembling specific aspects found in human retinal pathologies, demonstrating that AAVs are a valuable tool to generate unique insights into disease pathogenesis.

## Methods

### Molecular Cloning

Human DNA sequences of VEGF-A_165_, TNF-α, and IL-6 were codon optimized for human expression and a V5-tag was added at the C-terminus of each protein. Plasmids expressing human VEGF-A_165_ (hereafter referred to as VEGF), TNF-α, and IL-6 under the control of a ubiquitous CAG promoter were generated by gene synthesis (GeneArt, ThermoFisher). Sequences were sub-cloned into the pAAV plasmid (AAV Helper-Free System, Agilent) with a ubiquitous CAG promoter by Gibson assembly (NEB). To verify the Müller glia enriched infection of the ShH10 capsid, an eGFP expression construct under the control of the ubiquitous CMV promoter was used. As negative control, we used an AAV-stuffer construct, including fragments of the 3′ untranslated region of the *UBE3A* gene, as described previously.^45^ The sequence of AAV-stuffer is also shown in the Supplementary Material.

### AAV Production and Quantification

AAVs were produced using frozen high-density HEK293 cells in CELL discs and purified by polyethylenglycol precipitation and iodixanol gradient centrifugation according to previously published protocols.^46^ AAVs were dissolved in AAV formulation buffer (1 × PBS, 1 mM MgCl_2_, 2.5 mM KCl, 10% glycerol, 0.001% Pluronic F-68, pH 7.4, and sterile-filtered). All AAVs produced in this study were packaged into the ShH10 capsid.^2^ For AAV quantification, viral DNA was extracted, and quantitative polymerase chain reaction (PCR) was performed.^46^

### Animal Experiments

C57BL/6J mice were purchased from Charles River (Sulzfeld, Germany). Mice were housed in individually ventilated cages in groups of 2 to 5, constant temperature and a 12-hour light/dark cycle. Mice had ad libitum access to standard rodent chow and water. Then, the 6 to 8 week old male and female mice were used for all experiments. For in vivo imaging and IVT injection (described below), the mice were anesthetized with 60 to 90 mg/kg ketamine (Medistar) and 6 to 8 mg/kg xylazine (Rompun; Bayer) injected intraperitoneally. Mice were euthanized by cervical dislocation and whole eyes for expression analysis were snap-frozen in liquid nitrogen or fixed in 4% paraformaldehyde (PFA) for histology. Animal experiments were performed in accordance with the German Animal Welfare Act, the guidelines of the Federation of the European Laboratory Animal Science Association (FELASA), and the ARVO statement for the use of animals in ophthalmic and vision research. Animal experiments performed in this study were reviewed and approved by the local authorities.

### Intravitreal Injection

IVT injections were performed under ketamine/xylazine anesthesia (described above). Before IVT injection, local anesthesia was applied to the eyes (Novesin; OmniVision). Then, the eye was fixed with forceps and a 34-G needle was inserted peripheral to the limbus (the border between the cornea and the conjunctiva/sclera) through the pars plana. Then, the 1 × 10^8^ viral genomes (VG)/eye (low dose) or 1 × 10^9^ VG/eye (high dose) were injected. The negative control AAV-stuffer was injected at 4 × 10^9^ VG/eye. The IVT injection volume was 1 µL per eye. A total of 10 to 30 eyes were injected per AAV and animals were euthanized at 1, 3, or 6 weeks after injection. Eyes in which a major blood vessel or the lens was injured due to the IVT injection procedure were excluded from analysis.

### In Vivo Imaging

Mice were anesthetized as described above and pupils dilated with 5 mg/mL tropicamide (Mydrum; Bausch + Lomb) and phenylephrine (Neosynephrin-POS 10%; Ursapharm). Optical coherence tomography (OCT) analysis was performed with a Bioptigen Envisu R2210 device (Leica Microsystems) equipped with a lens specifically designed for mouse retinae (50 degrees field of view). Infrared Reflectance (IR) and Blue Autofluorescence (BAF) images were recorded with a Spectralis HRA/OCT device (Heidelberg Engineering) using a 55 degrees widefield lens. For fundus fluorescein angiography (FFA), 200 µL of a 0.2 % fluorescein solution (Alcon) was injected subcutaneously and pictures were recorded 90 seconds after injection with the Spectralis HRA/OCT device (Heidelberg Engineering). During image acquisition, mouse contact lenses (Heidelberg Engineering) were applied to the mouse eyes to prevent drying and improve the image quality. Fundus pictures were recorded with a Micron IV Fundus camera (Phoenix) equipped with a mouse-specific objective.

### Tissue Lysis and ELISA

Whole mouse eye lysates were generated by homogenization in ice-cold lysis buffer (9803; Cell Signalling) freshly supplemented with proteinase inhibitor Pefabloc SC (76307-100MG; Sigma) with metal beads (15987602; Bertin Technologies) in a Precellys Evolution tissue homogenizer (Bertin Technologies). After homogenization, samples were centrifuged at >16,000 g and 4°C for 10 minutes and the supernatant carefully collected. To measure human VEGF, TNF-α, or IL-6 in tissue lysates, DuoSet ELISAs (DY293B, DY210, DY206: RnD Systems) were used according to the manufacturer's manual. Samples were measured with a SpectraMax Plus 384 plate reader (Molecular Devices) and normalized to total protein concentration as measured by BSA protein assay (Pierce).

### Immunohistochemistry of Eye Cross-Sections

Eyes were enucleated and fixed in 4% PFA (AR1068; Boster) in phosphate-buffered saline (PBS) for at least 48 hours at 4°C. Tissues were dehydrated by incubation in stepwise increasing alcohol concentrations, incubated in xylol, and infiltrated with paraffin using a tissue processing machine (Tissue-Tek VIP 6; Sakura). Then, 3 µm sections were cut and hematoxylin and eosin (H&E) stainings were performed by TPL Path Labs (Freiburg, Germany). Masson Trichrome staining was done according to standard protocols.^47^ Immunohistochemical stainings were carried out on the automated Leica Bond platform (Leica Biosystems, Melbourne, Australia). Bound antibodies were visualized using the Bond Polymer Refine Detection System (Leica Biosystems, Newcastle, UK) or the Opal Multiplex IHC Kit (Akoya Biosciences). Depending on the antibody, antigen retrieval was either done with heat (95 degrees for 20 minutes) or proteolytic enzyme treatment (37°C for 5 minutes, BOND Enzyme pretreatment kit; AR9551). See Supplementary Table S1 for details on the antibodies and antigen retrieval used in this study. Spectral DAPI (FP1490; Akoya Biosciences) was used for nuclear staining and slides were mounted with ProLong Antifade Mounting Medium (P36961; Invitrogen). Slides were imaged using a laser-scanning microscope LSM710 (Carl Zeiss Microscopy GmbH) or an AxioScan.Z1 slide scanner (200 × magnification; Carl Zeiss Microscopy GmbH).

### Immunohistochemistry of Retinal and RPE/Choroid Flat Mounts

Eyes were enucleated and fixed in 4% PFA in PBS (AR1068; Boster) for 1 hour at room temperature. Retina and RPE/choroid were dissected with fine forceps and four radial cuts were made to flatten the retina. Tissues were blocked and permeabilized while slowly shaking at room temperature for 1 hour in 1 mL blocking buffer (0.5% Triton X-100 and 1% skimmed milk in PBS). Primary antibodies (see Supplementary Table S1) were diluted in blocking buffer and tissues were incubated in primary antibodies at 4°C overnight. After 3 washes with PBS, matching secondary antibodies labeled with a fluorophore were incubated at 4°C overnight. Nuclei were stained with 1:1000 diluted DAPI solution (62248; ThermoFisher). Tissues were washed three times in PBS. SecureSeal Imaging Spacer (SS8 × 9; Biotrend) were applied to glass slides and single retinae were transferred into each well of the spacer. Then, 8 µL Vectashield (Vectorlabs) was added to each tissue and sealed with a cover slip. Tissues were imaged with a laser-scanning microscope (LSM710; Zeiss) or an Opera Phenix High-Content Screening System (PerkinElmer).

### MSD Expression Analysis

A customized MSD U-Plex assay (Meso Scale Discovery) was used to measure 10 mouse cytokines and chemokines in parallel. The following proteins were analyzed: IFN-g, IL-1b, IL-4, IL-6, IL-10, TNF-α, IL-13, VEGF, MCP-1, and MMP9. Whole mouse eye lysates were diluted to 5 µg/mL total protein concentration. MSD assay was performed according to the manufacturer's protocol. SECTOR Imager 6000 (Mesoscale) was used for sample detection and quantification.

### Statistical Analysis

Statistical analysis was performed using GraphPad Prism. Individual statistical tests are mentioned in the respective figure legends. For comparison of multiple groups to a control group, 1-way ANOVA with Dunnett's post hoc test was used. Significance was determined according to the *P* value: *P < 0.05, **P < 0.01, ***P < 0.001, and ****P < 0.0001. Bar plot graphs represent the mean values ± SD.

## Results

### Generation and Characterization of AAVs Coding for Human VEGF, TNF-α, and IL-6

To produce AAVs encoding either human VEGF-A_165_ (VEGF), TNF-α, or IL-6, we cloned the respective transgenes into a plasmid driving expression via the ubiquitous CAG promoter. Plasmids then were subsequently packaged into the ShH10 virus capsid known to primarily transduce Müller glia.^2^ Müller glia have long extensions and span the full retina; therefore, we expect the secreted cytokines to be distributed throughout the retina. Non-coding stuffer DNA packaged into the same viral capsid (hereafter referred to as “AAV-stuffer”)^45^ was used as a control AAV. To validate transgene expression and function of our three transgenes, porcine retinal explants were transduced with AAV-VEGF, AAV-TNF-α, AAV-IL-6, or AAV-stuffer. Supernatants were collected during explant culture and proteins measured by ELISA (see Supplementary Fig. S1A). As expected, concentration of human VEGF (see Supplementary Fig. S1B), TNF-α (see Supplementary Fig. S1C), and IL-6 (see Supplementary Fig. S1D) in the supernatant was increased during retinal explant culture after transduction with the respective AAVs, verifying the production of the correct transgenes. Next, we tested for function of VEGF, TNF-α, and IL-6 by using the retinal explant supernatants in different activity assays. First, VEGF containing supernatants induced phosphorylation of VEGFR2 in human retinal microvascular endothelial cells (HRMECs; see Supplementary Fig. S1E), indicating that AAV-VEGF drives expression of functionally active VEGF. Moreover, AAV-produced TNF-α induced reporter gene expression in HEK Blue TNF-α cells (see Supplementary Fig. S1F) and AAV-produced IL-6 in HEK Blue reporter IL-6 cells (see Supplementary Fig. S1G). Altogether, we demonstrated that AAV transduction of porcine retinal explants led to stable expression of functionally active VEGF, TNF-α, and IL-6.

### Characterization of AAV-Stuffer as Control AAV and Transduction of Müller Cells Using AAV-GFP

We next evaluated the effects of our control AAV-stuffer on retinal morphology by IVT injection followed by in vivo imaging techniques, such as OCT, fundus analysis, and fundus fluorescein angiography (FFA; Supplementary Fig. S2). Retinal morphology of the AAV-injected eyes appeared normal as judged by OCT and FFA. However, we observed mild alterations in the fundus analysis, as bright areas of unknown origin were seen on some retinae. Because these bright areas were seen with all AAV injections in this study, we speculate that these are unspecific reactions toward the virus itself or the formulation buffer. We also tested two lower doses of AAV-stuffer and did not observe any inflammation or other side effects apart from the unspecific bright areas at any given dose (data not shown). Histological cross sections of eyes demonstrated an intact retinal morphology, regular vasculature, no fibrosis and inactive microglia/Müller glia (Supplementary Figs. S3A–D). Next, we validated the Müller glia enriched transduction by using an AAV-ShH10 construct expressing green fluorescent protein (AAV-GFP). Histological analysis indeed showed that mainly Müller glia expressed GFP, but also few ganglion cells or photoreceptor cells were GFP-positive (see Supplementary Fig. S3E). Overall, the AAV-stuffer had no major effect on the retinal morphology and AAV-GFP showed enriched transfection of Müller glia. Therefore, we concluded that the ShH10-based AAVs are suitable for expression of transgenes in the mouse retina and does not induce retinal pathologies on its own.

### Expression of Human VEGF, TNF-α, and IL-6 in the Murine Retina

**Figure 1.**
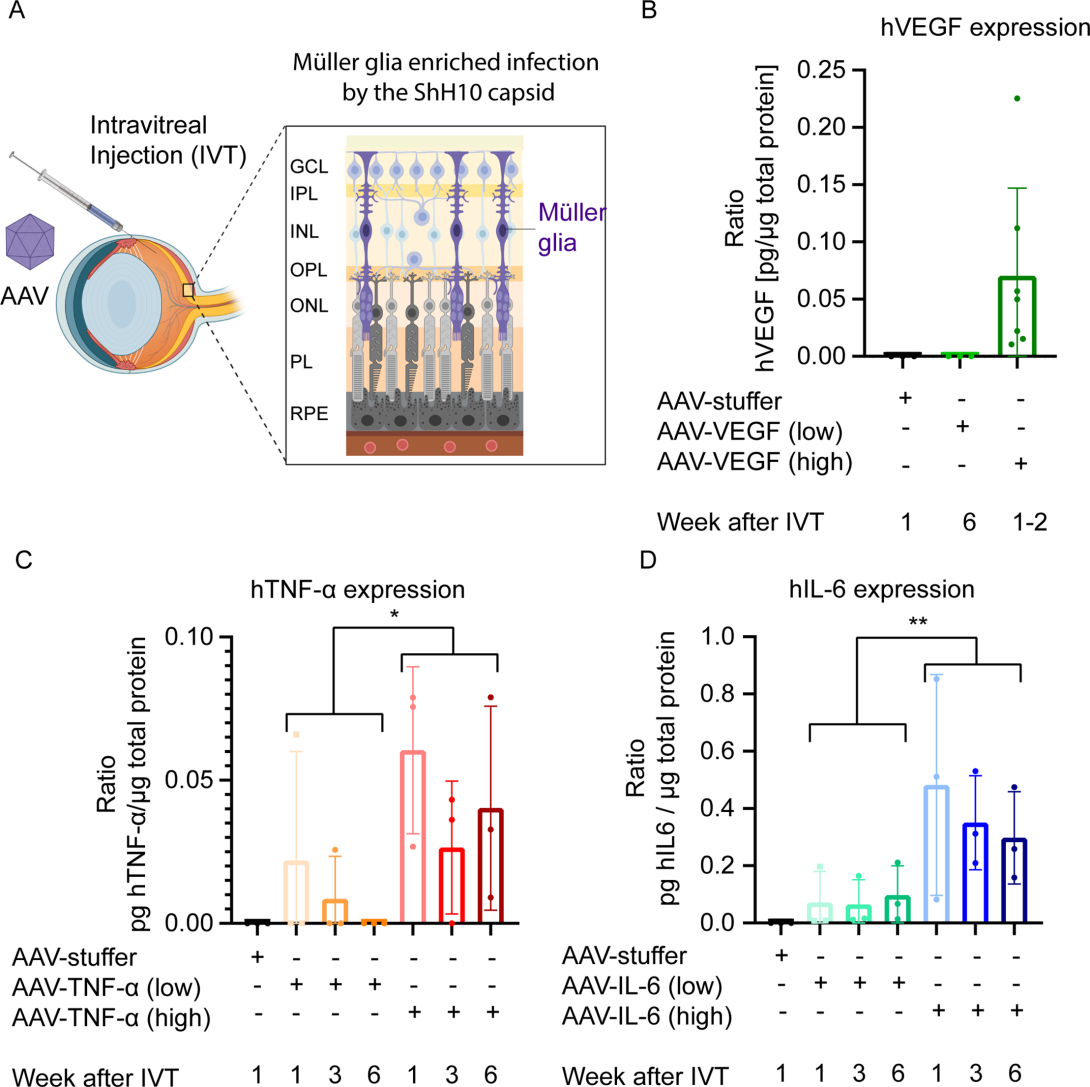
Expression of human VEGF, TNF-α, and IL-6 1 to 6 weeks after IVT injection of AAV-VEGF, AAV-TNF-α, and AAV-IL-6, respectively. (**A**) Experimental setup. AAVs with the ShH10-capsid are injected into the mouse vitreous to transduce Müller glia in the retina. (**B**) 0.07 pg VEGF/µg total protein was detected in whole mouse eye lysates by ELISA 1 to 2 weeks after IVT injection of high dose AAV-VEGF (*n* = 3–7). (**C**) 0.04 pg TNF-α/µg total protein was detected in whole mouse eye lysates 1 to 6 weeks after IVT injection of high dose AAV-TNF-α (*n* = 3). (**D**) 0.08 and 0.38 pg IL-6/µg total protein were detected in whole mouse eye lysates 1 to 6 weeks after IVT injection of low or high dose AAV-IL-6, respectively (*n* = 3). Low dose = 1 × 10^8^ VG/eye. High dose = 1 × 10^9^ VG/eye. Statistical analysis was done by 2-way ANOVA. Error bars represent the standard deviation (SD).

Having validated our experimental approach, we next aimed to express human VEGF, TNF-α, and IL-6 in the mouse eye. Therefore, we intravitreally injected two different doses of AAV-VEGF, AAV-TNF-α, AAV-IL-6, or AAV-stuffer and collected eyes 1, 3, or 6 weeks after injection (Fig. 1A). In low-dose AAV-VEGF injected eyes, VEGF expression was below the detection limit of the ELISA (<31.3 pg/mL VEGF), but high dose AAV-VEGF led to an exposure of approximately 0.07 pg VEGF per µg total protein (Fig. 1B). Notably, low-dose AAV-VEGF injected eyes were collected after 6 weeks, whereas high dose eyes were collected 1 to 2 weeks post IVT injection of the AAV-VEGF, due to the rapid onset of strong ocular pathologies (see below). Injection with high dose AAV-TNF-α, resulted in an exposure of 0.04 pg TNF-α per µg total protein, but low dose AAV-TNF-α did not lead to detectable TNF-α expression in most samples (Fig. 1C). Furthermore, approximately 0.08 pg IL-6 in low dose and 0.3 pg per µg total protein in AAV-IL-6 high dose injected eyes were detected (Fig. 1D). Altogether, high dose injection of AAV-VEGF, TNF-α, and IL-6 led to detectable and stable expression of the three respective transgenes.

### AAV-Mediated Expression of VEGF Leads to Vascular Pathologies in the Mouse Eye

**Figure 2.**
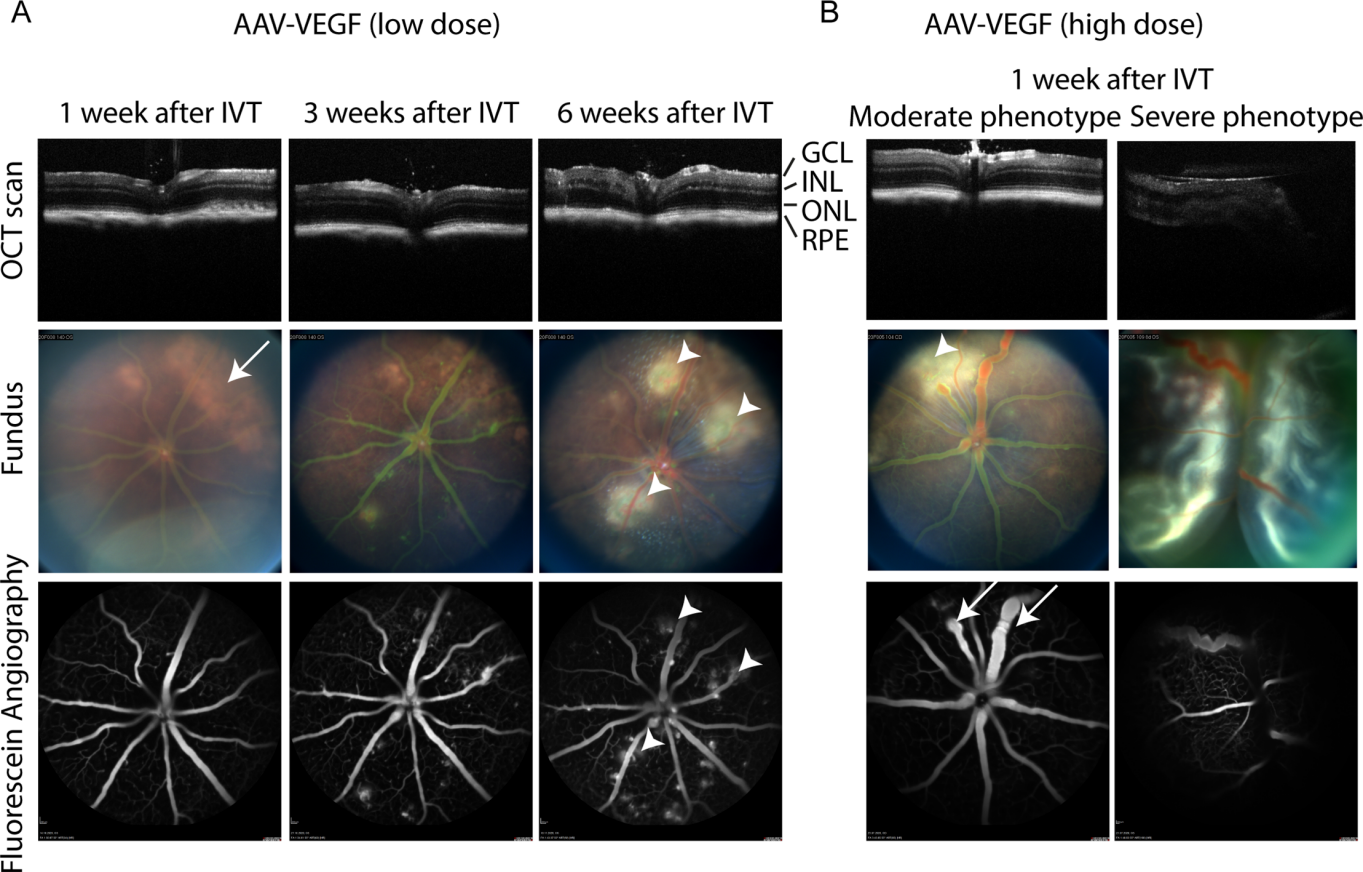
AAV-VEGF injection led to vascular pathologies in mouse eyes. (**A**) IVT injection of low dose AAV-VEGF led to progressive increase of vascular permeability starting from 3 weeks after injection as seen by FFA in the same mouse eye. White spots co-localizing the leakage areas were found by fundus imaging (*arrowheads*). Bright, unspecific areas were observed at earlier timepoints (arrow) that fainted over time and were also seen in AAV-stuffer control eyes (see Supplementary Fig. S2). (**B**) IVT injection of high dose AAV-VEGF led to focally dilated or constricted vessels (*arrow*) and vascular leakage 1 week after injection in 50% of the mice (“moderate phenotype,” *left column*). There was 50% of the mice that developed severe pathologies, including retinal detachment (*right column*). GCL = ganglion cell layer; INL = inner nuclear layer; ONL = outer nuclear layer; RPE = retinal pigment epithelium; low dose = 1 × 10^8^; high dose = 1 × 10^9^ VG/eye.

Six weeks after IVT injection of AAV-VEGF (low dose), we observed mild alterations in the OCT scan (i.e. irregular inner nuclear layer structure; Fig. 2, top row). Fundus pictures showed transient, unspecific bright areas on the retina 1 week after IVT injection of AAV-VEGF (Fig. 2A, white arrows) reminiscent to the effects seen with the control virus (see Supplementary Fig. S2). In contrast, we observed specific white spots next to blood vessels (see Fig. 2A, white arrowheads) that increased with time only in VEGF expressing eyes on fundus pictures, suggesting fluid extravasation from the vasculature. Indeed, FFA demonstrated that size and number of areas with fluorescein outside of the blood vessels increased with time in AAV-VEGF injected eyes (see Fig. 2A, bottom row, white arrowheads), suggesting that VEGF increased vascular permeability. Furthermore, we also analyzed mouse eyes after injection of a higher dose of AAV-VEGF. Of note, 14 of 28 eyes treated with high dose AAV-VEGF were clearly enlarged 1 to 2 weeks after injection preventing in most cases the recording of regular OCT or fundus images (Fig. 2B, right panel, “severe phenotype”). These eyes were fluid-filled, exhibited increased ocular pressure, and were easily damaged by enucleation (data not shown). Our data suggest that in these severely affected eyes, the retina was detached, complicating in vivo imaging. In contrast, non-enlarged eyes could be properly imaged and showed localized leakiness of vessels as well as enlarged or constricted vessels already 1 to 2 weeks after treatment (see Fig. 2B, left panel, white arrow). Altogether, injection of AAV-VEGF led to specific, dose-dependent vascular leakage in the murine eye.

### Histological Analysis of VEGF Expressing Eyes

**Figure 3.**
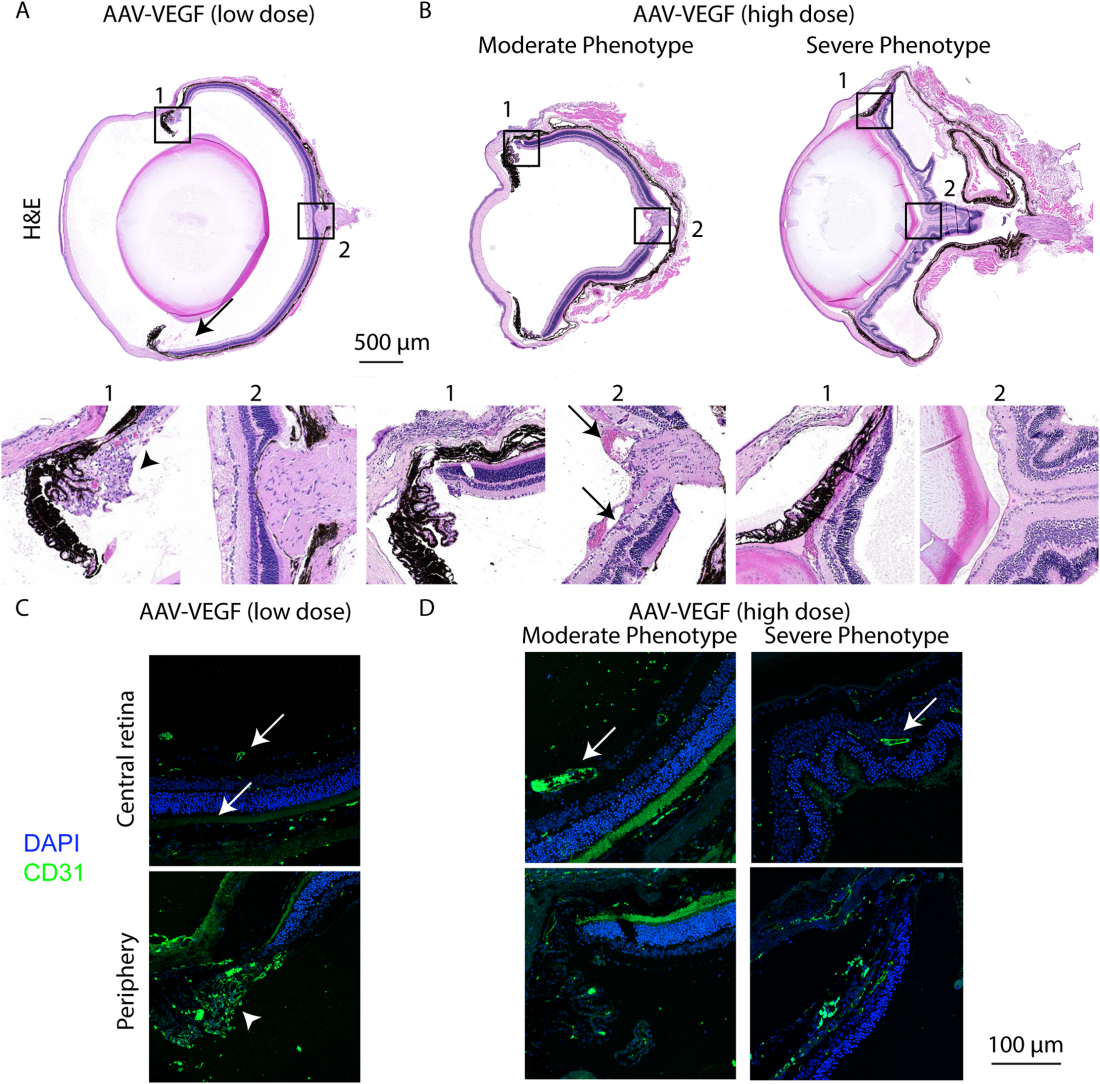
Neovascularization was observed in AAV-VEGF treated eyes. (**A**) Cross-sections of eyes injected with low dose AAV-VEGF revealed neovascularization (*arrowhead*) in the peripheral areas of the retina (zoom panel 1) and vitreous hemorrhage (*arrow*) 6 weeks after IVT injection. (**B**) High dose AAV-VEGF injection led to enlarged blood vessels (*arrow* zoom panel 2) but no neovascularization, in the moderately affected eyes. In contrast, retinal folding and retinal detachment was observed in severely affected eyes injected with high dose AAV-VEGF. (**C**) Immunostaining with endothelial cell marker CD31/PECAM (*green*) and DAPI (*blue*) showed mostly normal blood vessels in the GCL (on top), IPL, and OPL in the central area of AAV-VEGF (1 × 10^8^ VG/eye) injected eyes. CD31-positive staining in the far periphery (*white arrowhead*) verified that endothelial cells are present in this area. (**D**) Injection of high dose AAV-VEGF led to enlarged blood vessels (*white arrow*) in the inner retina and retinal folding in severely affected eyes (CD31 = *green*, DAPI = *b*lue). Low dose = 1 × 10^8^; high dose = 1 × 10^9^ VG/eye.

To further characterize the pathologies induced by AAV-VEGF, we next analyzed histological cross sections of the mouse eye. Six weeks after IVT injection of AAV-VEGF (low dose), erythrocytes were present in the vitreous (vitreous hemorrhage; Fig. 3A, black arrow), in accordance with our in vivo imaging data demonstrating increased vascular permeability (see Fig. 2). Strikingly, in far peripheral regions of the retina, we observed abnormal blood vessels growing into the vitreous and enlarged and irregular blood vessels within the retina (see Fig. 3A, insert 1, black arrowheads), indicating retinal neovascularization. Similarly, injection of the high dose affected the morphology of the vasculature already within 1 to 2 weeks, as shown by enlarged blood vessels and irregular vessel growth (Fig. 3B, left panel, black arrow). Only a few eyes injected with high dose AAV-VEGF presented neovascularization in the periphery, potentially due to the shorter disease progression time. Furthermore, the severely affected eyes not analyzable by our in vivo imaging techniques, presented massive pathologies including retinal folding and retinal detachment (see Fig. 3B, right panel). To verify abnormal endothelial cells proliferation, we used immunofluorescent staining of CD31/PECAM, a marker for endothelial cells. CD31-positive cells were found in the ganglion cell layer (GCL), inner plexiform layer (IPL), and outer plexiform layer (OPL), as expected. In AAV-VEGF injected eyes, blood vessels with enlarged diameter were found (Figs. 3C, 3D, white arrows) compared to control eyes (see Supplementary Fig. S3B). Additionally, the abnormally growing cells in the far periphery of AAV-VEGF (low dose) animals are indeed endothelial cells, as verified by positive CD31 staining. To sum up, histological analysis verified retinal detachment in severely affected eyes of high dose AAV-VEGF. Moreover, we demonstrated that injection of a lower dose AAV-VEGF induces retinal neovascularization in the peripheral areas of the mouse eye 6 weeks after IVT injection.

### AAV-Mediated Expression of TNF-α Induces Inflammation in the Mouse Eye

**Figure 4.**
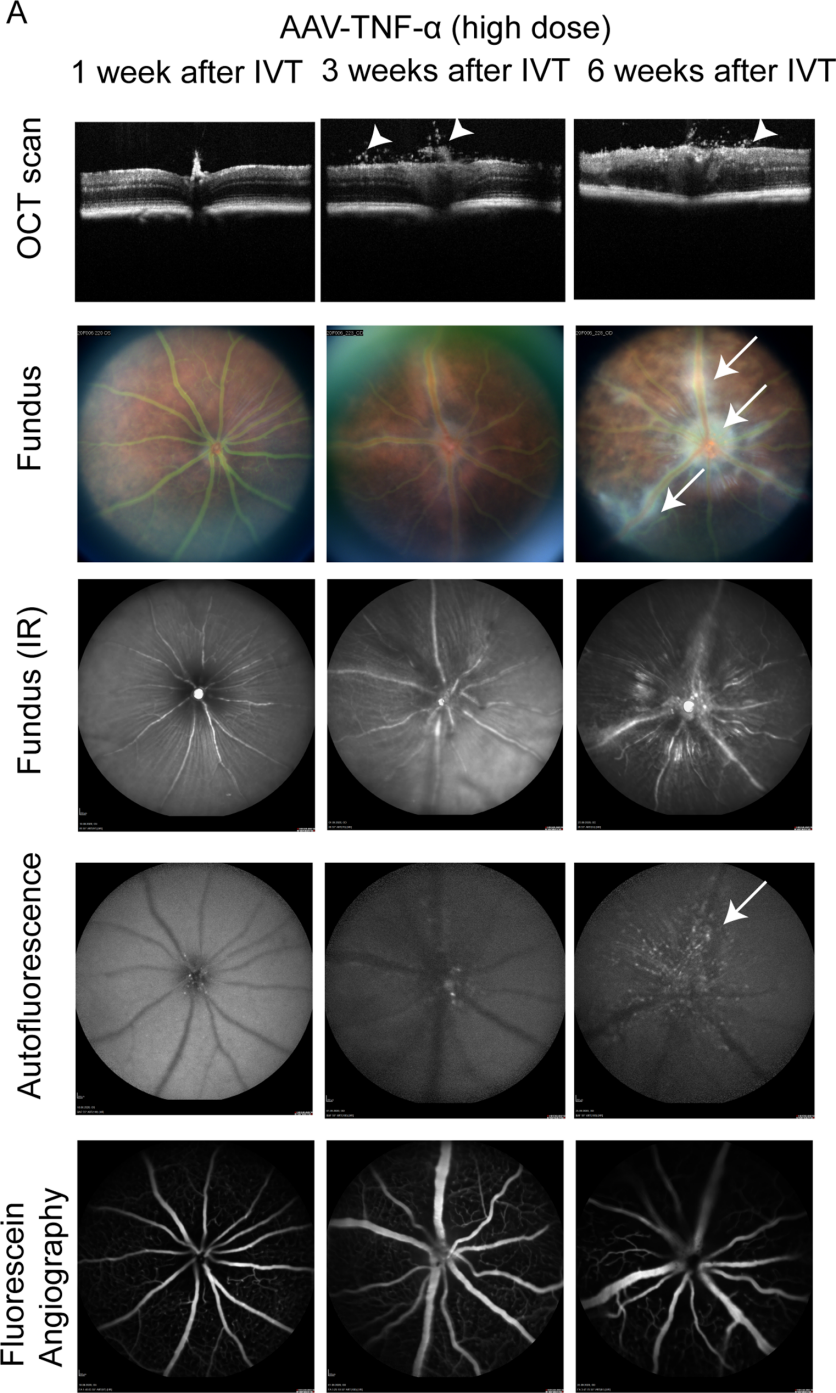
Inflammation was observed in AAV-TNF-α injected eyes. (**A**) OCT scans revealed cellular infiltrates in the vitreous (*white arrowheads*) 3 to 6 weeks after injection of AAV-TNF-α (*upper row*). White cellular infiltrates were observed around the vasculature and the optic nerve on fundus pictures (*second and third row, arrows*) in AAV-TNF-α injected eyes. Few hyperfluorescent spots were found around the optic nerve and vasculature in AAV-TNF-α injected eyes by BAF. FFA was largely normal in AAV-TNF-α eyes. Different animals were examined at 1, 3, and 6 weeks after IVT. 1 × 10^9^ VG/eye AAV-TNF-α was injected intravitreally.

Next, we injected AAV-TNF-α into the mouse eye to express the human proinflammatory cytokine TNF-α. Administration of 1 × 10^8^ VG/eye (low dose) did not induce any phenotype at 1, 3, or 6 weeks after IVT injection (data not shown). In contrast, 3 weeks after IVT treatment of high dose AAV-TNF-α, we observed cellular infiltrates in the vitreous and the optic nerve appeared swollen in the OCT scans (Fig. 4A, middle column, white arrowheads). Additionally, the fundus of TNF-α expressing mice showed white cellular infiltrates around the optic nerve and the vasculature, indicating retinal vasculitis (white arrows). Few hyperfluorescent spots were observed in the central area of the retina as seen by Blue Autofluorescence (BAF) analysis (white arrows). Vasculature morphology or permeability appeared normal as seen by FFA. All observed phenotypes were stronger 6 weeks after IVT injection with partially or fully disorganized retinal layers and increased perivascular infiltrates (see Fig. 4A, right column). In contrast, we did not observe any inflammation-related phenotypes in AAV-stuffer injected control eyes (see Supplementary Fig. S2). Overall, AAV-mediated expression of TNF-α induced inflammation in the mouse eye, as seen by cellular infiltrates in the vitreous and retinal vasculitis.

### Histological Analysis of TNF-α Expressing Eyes

**Figure 5.**
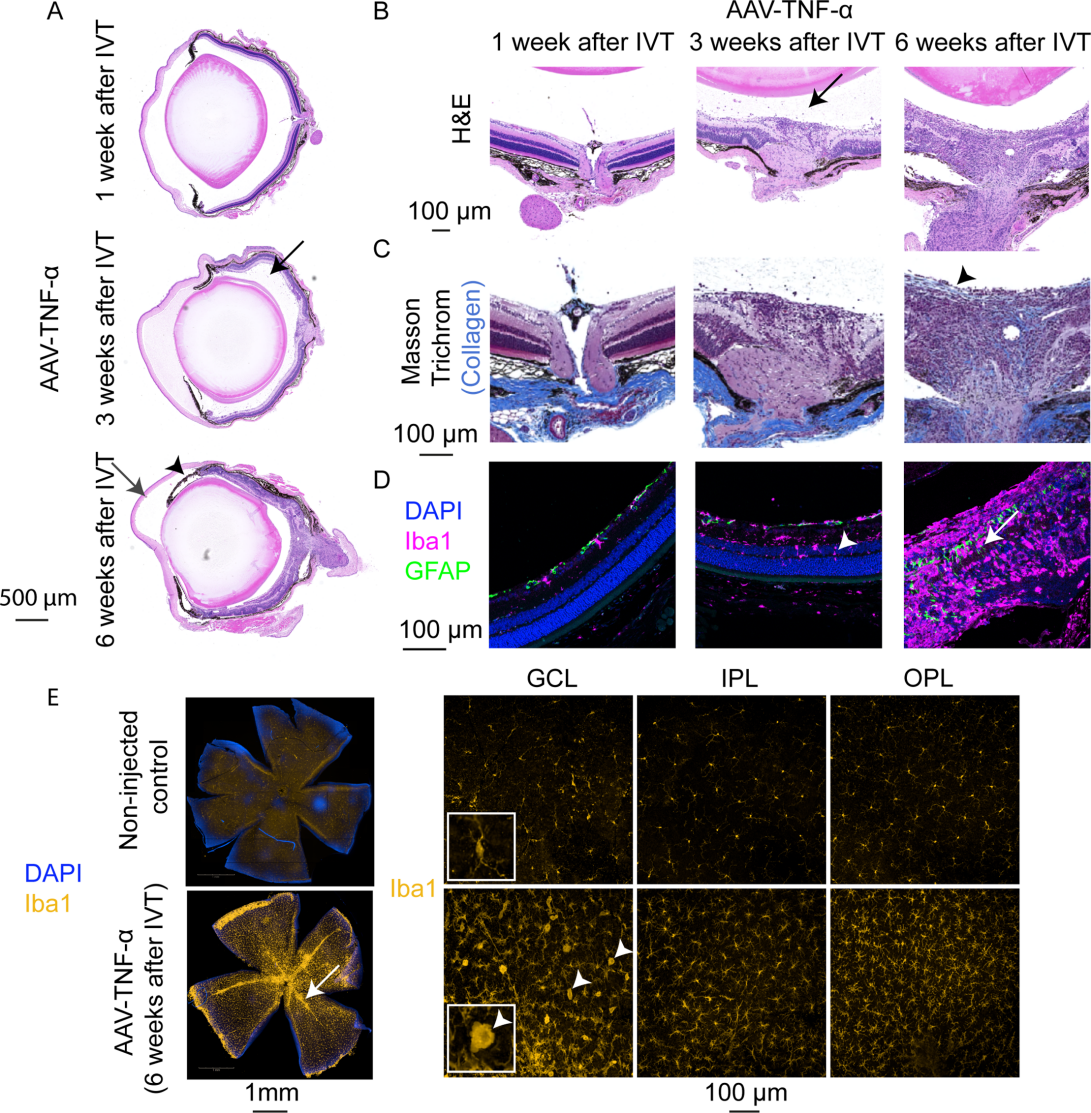
Immune cell activation and fibrosis in AAV-TNF-α injected eyes. (**A**) H&E staining of cross-sections of AAV-TNF-α injected eyes confirmed the presence of immune cell infiltration in the vitreous (*black arrow*) 3 weeks after IVT. Six weeks after IVT of AAV-TNF-α, retinal layers were largely disorganized, immune cells within the anterior chamber (*grey arrow*) and posterior synechiae (*black arrowhead*) were observed. (**B**) Zoom-in on the optic nerve of H&E stained eyes injected with AAV-TNF-α (see **A** for overview) with cellular infiltrates present in the vitreous (*black arrow*). (**C**) Collagen (*light blue*) was present within the retina in the area around the optic nerve and a newly developed epiretinal membrane (*black arrowhead*) in AAV-TNF-α injected eyes (Masson Trichrome staining). (**D**) Iba1 (*pink*), GFAP (*green*), and DAPI (*blue*) co-immunostaining revealed microglia/macrophage activation starting from 3 weeks after IVT injection of AAV-TNF-α, as indicated by the higher number of Iba1^+^ cells and the presence of Iba1^+^ cells within the nuclear layers (*white arrowhead*). Six weeks after IVT injection of AAV-TNF-α, GFAP^+^ Müller glia processes were observed across the retina (*white arrow*), indicating reactive gliosis. Note that pictures 6 weeks after IVT were recorded with a lower laser power due to the higher overall signal. (**E**) Retinal flat mounts stained with Iba1 (*yellow*) demonstrated the accumulation of Iba1^+^ cells around blood vessels (*left panel*, *white arrow*). As indicated by the higher magnification pictures (*right side*), the number of Iba1^+^ cells was increased in all three layers (GCL, IPL, and OPL) compared to the non-injected control retinae (*top row*) and many amoeboid shaped Iba1^+^ cells were present in the GCL (*white arrowhead*). The insert shows a zoom-in to better visualize the morphology of the Iba1^+^ cells in the GCL.

To further understand the pathophysiological changes in AAV-TNF-α treated mice, we analyzed histological cross sections of these eyes. H&E staining (Figs. 5A, 5B) verified cellular infiltrates in the vitreous (black arrows), as already seen by OCT 3 weeks after IVT injection of AAV-TNF-α. Then, 6 weeks after IVT, the inflammation-related phenotypes became more pronounced with partially or fully de-organized retinal layers. Fifty percent of the eyes also presented immune cells in the anterior chamber, largely de-organized retinal layers and the iris became adherent to the lens (posterior synechiae, black arrowheads). Since cytokines including TNF-α are well-known mediators of fibrosis,^48^ we also performed Masson Trichrome staining (see Fig. 4C) to investigate the presence of collagen within the eye. In control eyes (see Supplementary Fig. S3C) or 1 to 3 weeks after IVT treatment of AAV-TNF-α (Fig. 5C), collagen was only present in the sclera, as expected. In contrast, collagen was detected within 50% of the TNF-α expressing eyes 6 weeks after IVT injection around the optic nerve. Collagen was also present on a layer in the very inner region of the retina (see Fig. 5C, right panel, black arrowhead) reminiscent of epiretinal membranes developing in uveitis patients during chronic inflammation.^49^ Eyes were next stained by Iba1 and GFAP antibodies to investigate immune cell activation and reactive gliosis. Indeed, 3 weeks after IVT injection of AAV-TNF-α, increased number of Iba1^+^ microglia/macrophages were found and partially localized within the nuclear layers (Fig. 5D, white arrowhead), suggesting immune cell activation. Furthermore, 6 weeks after IVT treatment, the retina was largely disorganized and Iba1 staining was highly increased across the full retina. Moreover, at weeks 1 to 3 only astrocytes were labeled by GFAP, but 6 weeks after IVT of AAV-TNF-α Müller glia were active and labeled by GFAP as indicated by the cellular processes spanning across the retina (see Fig. 5D, white arrow). Finally, Iba1^+^ cells were specifically localized around the vasculature (Fig. 5E, white arrow) in retinal flatmounts, suggesting that the white cellular infiltrates seen in the fundus pictures were indeed immune cells (compare to Fig. 4A). As already detected in the cross sections, higher number of Iba1^+^ cells were observed in GCL, IPL, and OPL. Especially in the GCL, many microglia/macrophages were activated, as indicated by their rounder, amoeboid shape, in contrast to the non-injected control mice that presented largely inactive microglia (see Fig. 5E, white arrowheads). To sum up, histological analysis verified strong inflammation in TNF-α expressing eyes as seen by cellular infiltrates into the vitreous, active microglia/macrophages, reactive gliosis, fibrosis, and development of an epiretinal membrane.

### AAV-Mediated Expression of IL-6 Induces Inflammation in the Mouse Eye

**Figure 6.**
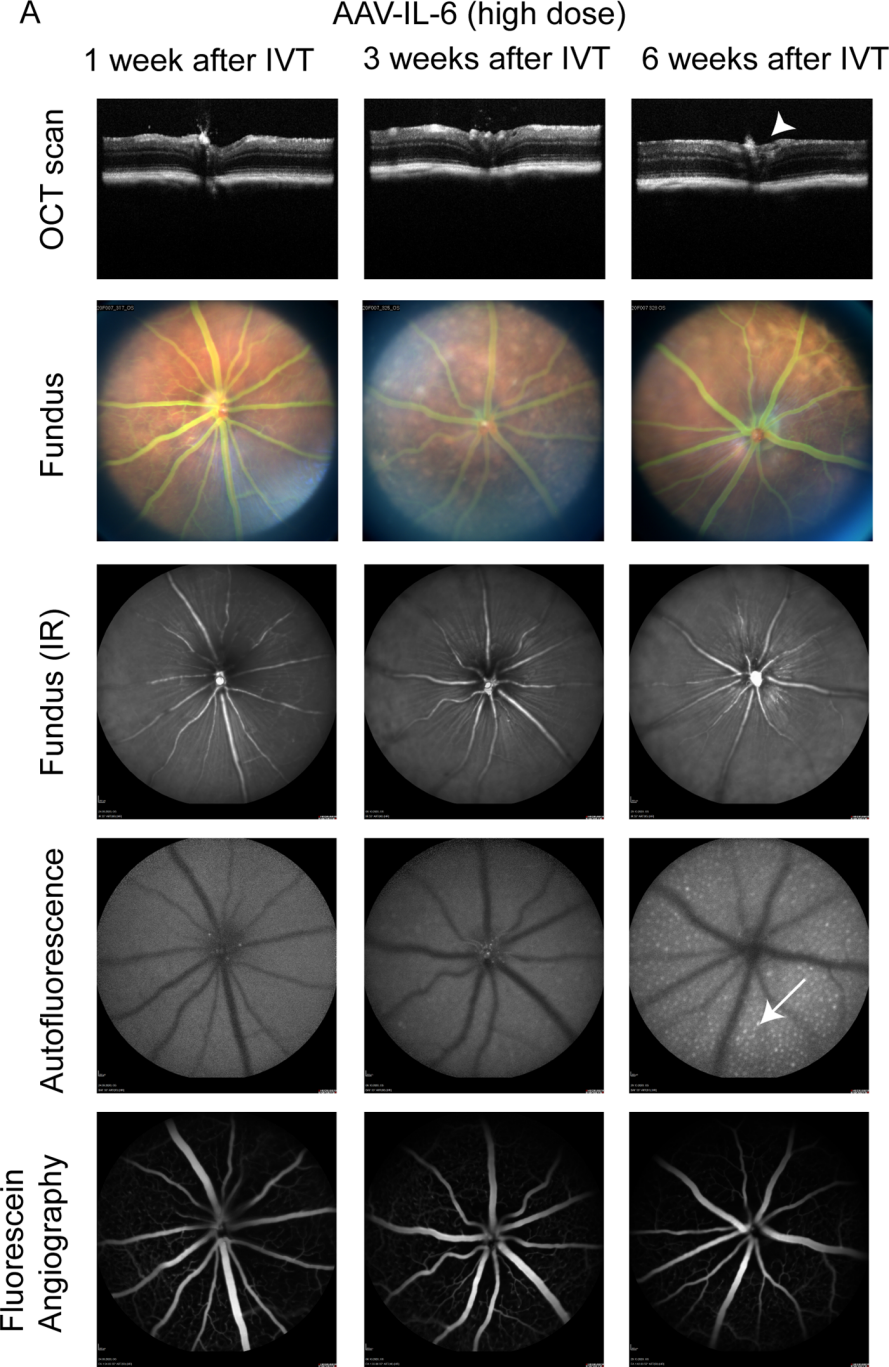
AAV-IL-6 injected eyes develop subretinal hyperfluorescent foci within 6 weeks. (**A**) OCT scans showed normal retinal morphology with slightly swollen/enlarged optic nerve (*arrowhead*). Fundus pictures appeared mostly normal. Blue autofluorescence measurements demonstrated hyperfluorescent foci (*arrow*) at the subretinal layer 6 weeks after IVT injection of AAV-IL-6. No vascular leakage was observed by FFA. Different animals were analyzed 1, 3, and 6 weeks after IVT. 1 × 10^9^ VG/eye AAV-IL-6 were injected IVT.

Finally, we evaluated pathologies induced by IL-6 in the murine eye. Mild swelling of the optic nerve was observed in AAV-IL-6 injected eyes 3 to 6 weeks after IVT treatment (Fig. 6A, top row, white arrowhead). The fundus pictures appeared relatively normal, apart from few bright lesions on the retina. FFA did not show any leakiness or vessel growth. However, we noticed smaller arteries and larger veins after AAV-IL-6 injection compared to baseline assessments in the same animals (data not shown). Most interestingly, we observed subretinal hyperfluorescent foci evenly distributed across the eye starting 3 to 6 weeks after IVT treatment with AAV-IL-6 (see Fig. 6, white arrows), indicating changes in the subretinal space that are often correlated with macrophage infiltration.^50^ Similar phenotypes were observed with both low dose (data not shown) or high dose (see Fig. 6) AAV-IL-6, but not in AAV-stuffer injected control eyes (see Supplementary Fig. S2). Altogether, injection of AAV-IL-6 led to mild swelling of the optic nerve and accumulation of hyperfluorescent foci in the subretinal space.

### Histological Analysis of IL-6 Expressing Eyes

**Figure 7.**
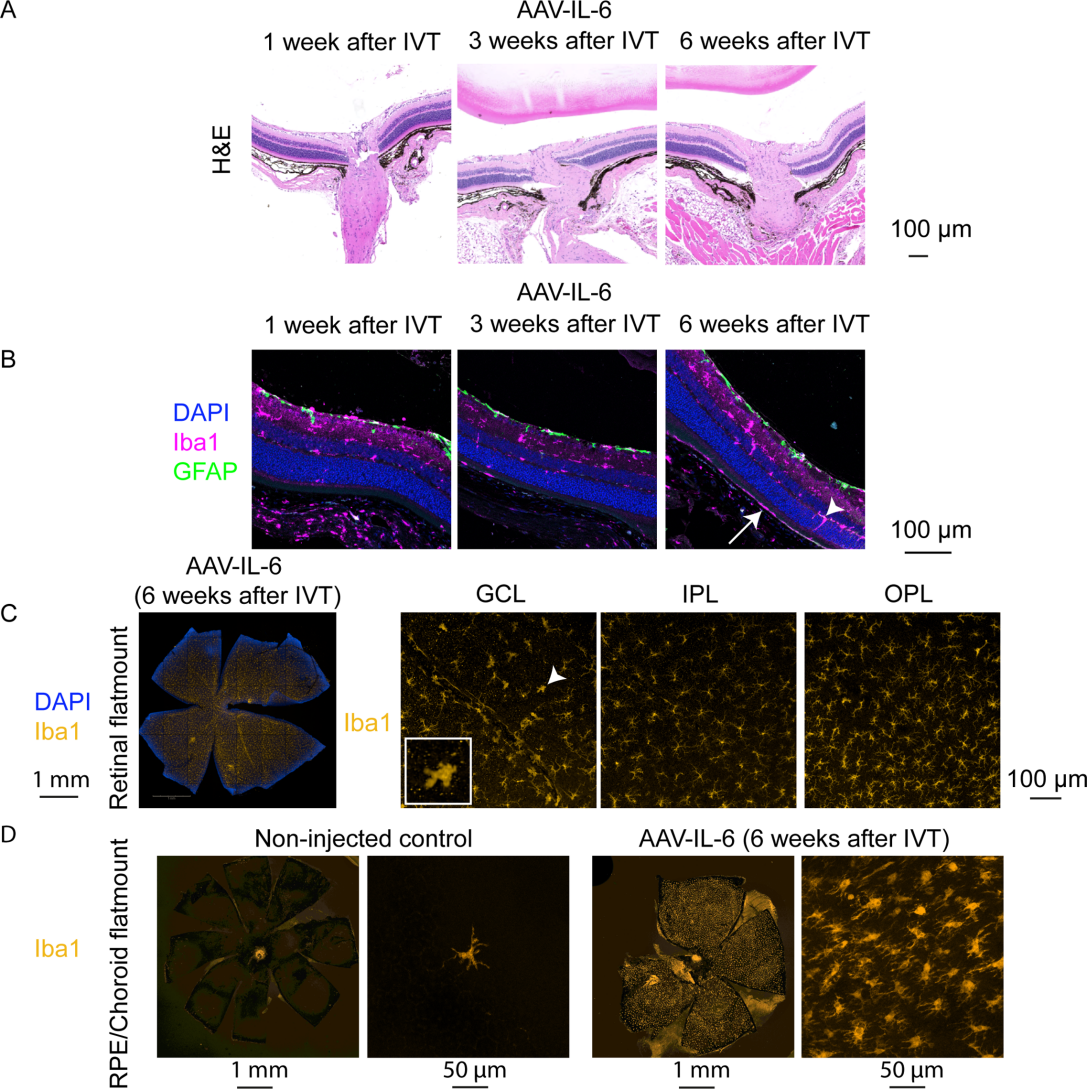
Iba1^+^ immune cells were activated and infiltrated the subretinal space of AAV-IL-6 injected eyes. (**A**) H&E stainings demonstrated a largly normal morphology in AAV-IL-6 retinae. (**B**) Co-immunostaining with Iba1 (*pink*), GFAP (*green*), and DAPI (*blue*) showed Iba1^+^ cells in within the nuclear layers (*arrowhead*) and subretinal (*arrow*), but no Müller glia activation (GFAP). (**C**) High number of Iba1^+^ cells (*yellow*) in the GCL, IPL, and OPL of the retina 6 weeks after IVT injection of AAV-IL-6 in retinal flat mounts, including Iba1^+^ cells in the amoeboid shape (*arrowhead*). (**D**) RPE/choroid flat mounts demonstrated only few Iba1^+^ immune cells (*yellow*) in RPE/choroid of non-injected control eyes (*left*), but high number of Iba1^+^ cells in AAV-IL-6 injected eyes 6 weeks after IVT treatment (*right*).

Cross-sections of IL-6 expressing eyes verified that the morphology was largely normal 1 to 6 weeks after IVT (Fig. 7A). GFAP immunostaining demonstrated absence of reactive gliosis at any time point. In contrast, activation of microglia/macrophages was observed at all time points as seen by the higher number of Iba1^+^ cells within the nuclear layers (white arrowhead) and the subretinal space (white arrow) starting from 3 weeks after IVT treatment in IL-6 expressing eyes (Fig. 7B). Similarly, retinal flat mounts stained with an Iba1 antibody indicated activation of microglia/macrophages 6 weeks after IVT injection of AAV-IL-6 (Fig. 7C, white arrowhead). Finally, because we observed hyperfluorescent foci in the subretinal layer in the BAF in vivo analysis (see Fig. 6) and these foci are reported to correlate with immune cells containing autofluorescent material,^51^ we stained RPE/choroid flat mounts with the Iba1 antibody. Strikingly, whereas in non-injected control mice only very few Iba1^+^ cells are present on the RPE, AAV-IL-6 RPEs were completely covered with Iba1^+^ immune cells (Fig. 7D). Altogether, we observed inflammation in IL-6 expressing eyes as seen by immune cell activation and infiltration into the subretinal space.

### Molecular Characterization of VEGF, TNF-α, and IL-6 Expressing Eyes

**Figure 8.**
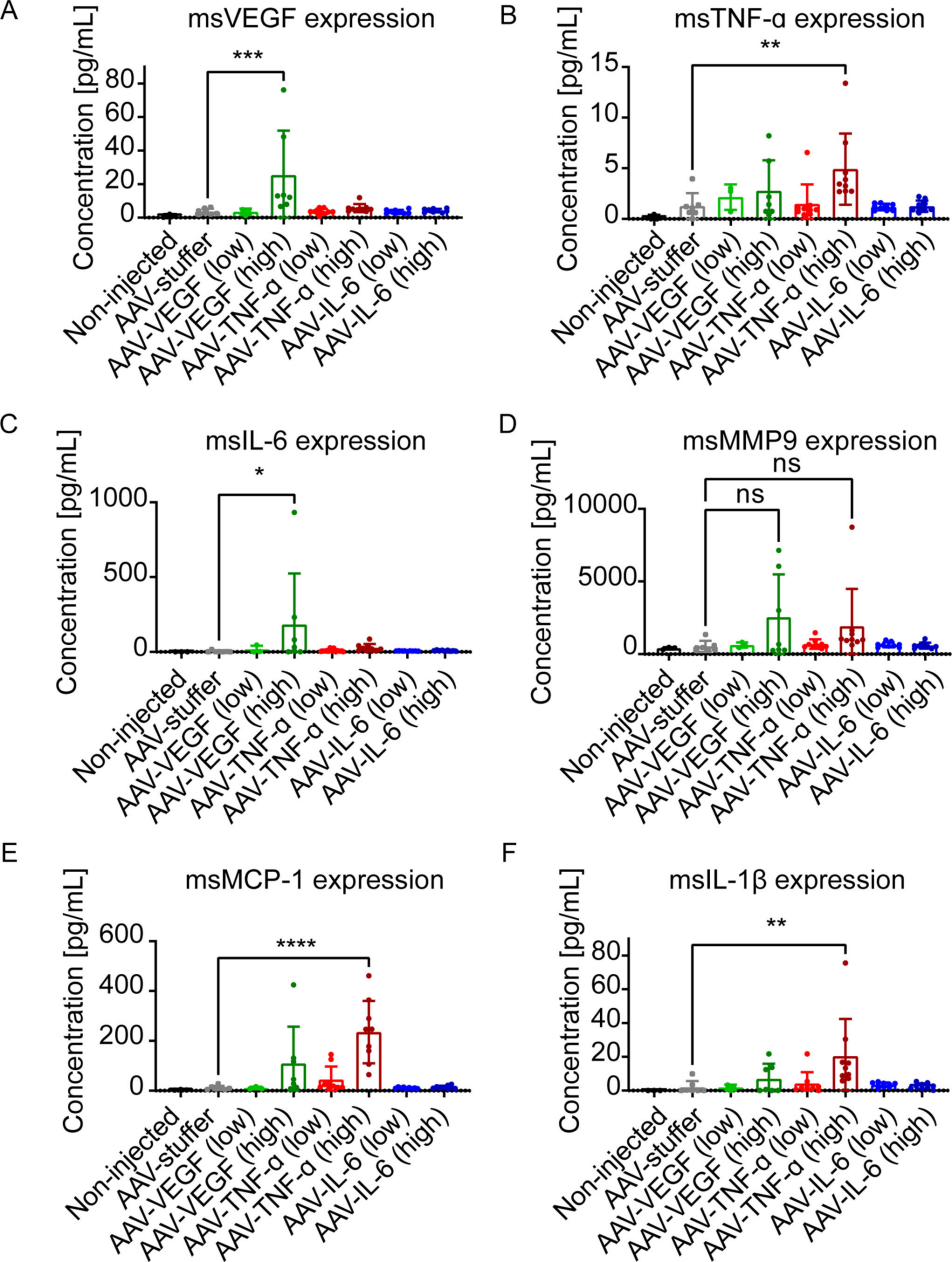
Expression analysis of mouse VEGF, TNF-α, IL-6, MMP9, MCP-1, and IL-1β in AAV-injected mouse eyes 1 to 6 weeks after IVT injection. (**A**) The msVEGF was 10-fold increased in hVEGF-expressing eyes, but not hTNF-α or hIL-6 expressing eyes compared to AAV-stuffer treated control eyes (***P < 0.001). (**B**) msTNF-α was 5 times higher expressed in hTNF-α expressing eyes (**P < 0.01) compared to AAV-stuffer. (**C**) The msIL-6 was higher expressed in AAV-VEGF-treated eyes (*P < 0.05). (**D**) MMP9 was not significantly changed in any condition tested. (**E**) MCP-1 expression was 11-fold induced by high dose of AAV-TNF-α (****P < 0.0001) compared to AAV-stuffer treated eyes. (**F**) IL-1β expression was 10-fold higher in AAV-TNF-α injected eyes compared to AAV-stuffer treated eyes. Statistical analysis was done by 1-way ANOVA followed by Dunnett's post hoc test (AAV-stuffer as control). Low dose = 1 × 10^8^; high dose = 1 × 10^9^ VG/eye.

To obtain further insight into the molecular mechanism underlying the observed phenotypes, we used a multiplex assay to measure relevant mouse cytokines and chemokines in mouse eye lysates. First, we tested if mouse VEGF (msVEGF) is induced by hVEGF, hTNF-α, or hIL-6. The msVEGF was only increased in eyes injected with AAV-hVEGF (high dose; Fig. 8A), but not in any other condition. Similarly, msTNF-α was only increased in eyes injected with a high dose of AAV-hTNF-α (Fig. 8B). Notably, antibodies used in the MSD kit are supposed to be mouse-specific, but a low degree of cross-reactivity cannot be excluded. Expression of msIL-6 was increased in AAV-VEGF treated eyes (Fig. 8C), suggesting that VEGF can induce IL-6 in mice. Next, we analyzed the expression of Matrix metallopeptidase 9 (MMP9), a well-known mediator of extracellular matrix breakdown and angiogenesis upregulated in patients with diabetic retinopathy and rodent models.^52^ There was a slight, but very variable increase in MMP9 in AAV-VEGF (high dose) and AAV-TNF-α (high dose) treated eyes, however, this was not statistically significant. In contrast, there was a strong and significant increase in expression of Monocyte chemoattractant protein-1 (MCP-1), a chemokine that regulates migration and infiltration of monocytes/macrophages, in AAV-TNF-α (high dose) injected eyes, but not in any other tested condition. This result confirms the strong increase and localization of Iba1^+^ microglia/macrophages specifically in TNF-α expressing eyes seen by immunohistochemistry (see Fig. 5E). Finally, we also analyzed expression of interleukin 1-β (IL-1β), a cytokine produced by activated macrophages. In line with the increase of MCP-1 expression, IL-1β expression was also increased in AAV-TNF-α (high dose), verifying a role for TNF-α in monocyte/macrophage recruitment and activation. To sum up, our MSD analysis confirmed a strong inflammatory response involving macrophage infiltration and activation specifically in TNF-α expressing eyes.

## Discussion

The aim of our study was to develop easy-to-use inducible mouse models of retinopathies using AAV mediated gene transfer. First, we expressed VEGF in the mouse eye by IVT injection of AAV-VEGF. Akin to the different VEGF-expressing transgenic lines generated by Lai et al., severity of vascular leakage correlated with the achieved expression levels of VEGF.^16^ Half of the eyes injected with the highest dose AAV-VEGF were enlarged and presented severe retinal folding and retinal detachment, comparable to transgenic mouse lines with very high expression of VEGF^16^^,^^18^ or the IGF1 transgenic mouse.^53^ Importantly, retinal detachment is a severe complication in patients with late stage proliferative DR.^54^ Additionally, we observed abnormal intraretinal growth of blood vessels as well as enlarged vessels, comparable to transgenic VEGF-expression models developing intraretinal neovascularization.^16^^,^^19^^,^^55^ Choroidal neovascularization is a hallmark of proliferative DR, however, in agreement with the findings in the transgenic Kimba VEGF mice, choroidal neovascularization was not observed. It has been speculated before that VEGF expression alone (from retina or RPE cells) is not sufficient to induce choroidal neovascularization due to an intact Bruch's membrane.^56^ In line with this hypothesis, choroidal neovascularization has been observed in several models that use subretinal injection of adenoviruses or AAVs expressing VEGF,^17^^,^^21^^,^^57^ suggesting that the process of the subretinal injection itself damages the Bruch's membrane and may contribute to the development of choroidal neovascularization. In our mice injected with AAV-VEGF, we observed neovessels growing into the vitreous. These vessels resemble neovascular tufts observed in patients with retinopathy of prematurity (ROP) and the corresponding animal model of oxygen induced retinopathy (OIR)^58^ as well as the DR-like IGF1 transgenic mouse model.^53^ Another feature of proliferative DR is vitreous hemorrhage,^59^ which was also observed in the AAV-VEGF transduced eyes. Because new vessels are often more fragile and therefore prone to rupture, we speculate that VEGF-induced neovascularization in AAV-VEGF injected eyes may contribute to vitreous hemorrhage. Altogether, we noticed similarities (e.g. vascular leakage) but also different pathologies (e.g. neovessels in vitreous) between our AAV-based approach and previously generated transgenic VEGF mice. One possible explanation for these differences might be the use of a truncated mouse rhodopsin promoter, inducing human VEGF-A expression between 5 and 10 days after birth in the Kimba mice,^55^ when the retinal vasculature is not yet fully developed, whereas the AAVs in our study were injected into the vitreous of fully developed, adult mice. Because AAVs may also be injected to other tissues, vascular pathologies in other organs, such as cerebral cavernous malformations,^60^ could in the future also be studied with the help of our VEGF-expressing AAV. Taken together, our easy-to-use AAV-based VEGF mouse model resembles several pathologies characteristic of proliferative DR or ROP in animal models and patients, including vascular leakage, neovascularization, and vitreous hemorrhage.

Next, we injected AAVs expressing TNF-α into the mouse vitreous and observed severe inflammation in the mouse eye. Only few reports describe a direct function of TNF-α in the retina of animal models. For example, IVT injected recombinant TNF-α leads to microglia activation in the optic nerve within 24 hours and RGC death 8 weeks after IVT treatment.^29^ In our study, we also observed activation of microglia/macrophages, not only localized to the optic nerve but rather across the retina and especially around the vasculature. Da Cunha et al. also used AAVs to express murine TNF-α in the mouse eye and observed cellular infiltrates in the vitreous and disorganized retinal layers 5 weeks after IVT of 5 × 10^8^ VG/eye AAV-TNF-α as well.^32^ In our study, we further demonstrate that also human TNF-α can induce such inflammation, as characterized by infiltrates in the vitreous and vasculitis and – at a later stage – fibrosis and epiretinal membranes. Infiltrates in the vitreous and vasculitis are also a common phenotype in patients and mouse models of uveitis, as, for example, observed in the experimental autoimmune uveoretinitis (EAU) model.^61^ In patients, epiretinal membranes often develop secondary to a pre-existing retinopathy, such as proliferative DR, PVR, and uveitis.^62^ Only few rodent models that develop epiretinal membranes have been introduced. For example, mice that overexpress Platelet-derived growth factor (PDGF) develop epiretinal membranes and retinal detachment.^63^ Finally, multiplex analysis of relevant cytokines revealed that expression of MCP-1 and IL-1β was induced in TNF-α expressing mice. MCP-1 and IL-1β are both well-known mediators of monocyte attraction and activation. Thus, these results fit to the increase and activation of Iba1^+^ microglia/macrophages observed by immunostainings of retinal flatmounts. Altogether, we demonstrated that TNF-α expression is sufficient to induce retinal inflammation as seen by immune cell infiltrates in the vitreous and vasculitis. In a second step, chronic inflammation led to reactive gliosis, fibrosis, and development of an epiretinal membrane, reflecting very well different stages of proliferative retinopathies or uveitis in patients.

Finally, we expressed IL-6 in the mouse eye by IVT injection of AAV-IL-6. Previous reports about the effects of increased IL-6 level in the mouse eye (by IVT injection of recombinant IL-6 or AAV-IL-6) are controversial: Some reports note inflammation in the eye caused by IL-6,^38^^,^^39^ whereas others found no inflammation.^32^^,^^40^^,^^41^ In our study, we observed an increase in microglia/macrophage numbers and activation across the retina and invasion of Iba1^+^ macrophages into the subretinal space / RPE, as described in another study injecting recombinant IL-6 into the eye.^39^ Only very few macrophages are usually present in the subretinal space of healthy mice, whereas macrophages accumulate there during ageing and in diverse retinopathy mouse models.^50^^,^^51^^,^^64^^–^^66^ For example, subretinal Iba1^+^ cells increase during aging and contain lipofuscin granules that are also visible as hyperfluorescent foci by BAF analysis^50^ comparable to our study. Overall, the inflammation induced by IL-6 was milder compared to the TNF-α induced inflammation, because we did not observe any signs of reactive gliosis or fibrosis with IL-6. This observation is in line with our multiplex analysis and previous results, suggesting that TNF-α is upstream in the inflammation cascade and able to induce other cytokines, in contrast to IL-6 that may not be sufficient to trigger a strong inflammatory response on its own.^32^ However, we only studied mice 6 weeks after IVT treatment with AAV-IL-6, due to the subtle effects of IL-6, it might take longer to see severe inflammation or reactive gliosis. Altogether, AAV-induced expression of IL-6 led to mild inflammation, including infiltration of macrophages into the subretinal space.

In conclusion, AAV-mediated expression of human VEGF, TNF-α, and IL-6 in the retina led to specific pathologies in the mouse eye, demonstrating that AAVs are a versatile tool to study the function of different proteins in vivo*.* It will be interesting to expand these observations by evaluating the retinal overexpression of other cytokines, investigating additional preclinical species to uncover potential species-specific effects, as well as to analyze a treatment-like setting upon the occurrence of AAV-induced retinal pathology. Furthermore, there is the potential to administer a combination of AAVs expressing different transgenes, allowing for their simultaneous expression upon a single IVT injection, which has the potential to resemble multiple features observed in human pathology. Overall, these data indicate that the AAV-mediated expression of transgenes will be a valuable tool to increase our understanding of disease pathology and explore novel treatment options for retinal diseases.

## Supplementary Material

Supplement 1
