# Supplementary material for: AAV-Mediated Expression of Human VEGF, TNF-α, and IL-6 Induces Retinal Pathology in Mice
Source: Transl Vis Sci Technol. 2021 Sep 14;10(11):15. doi: 10.1167/tvst.10.11.15 (PMC8444492; doi:10.1167/tvst.10.11.15)
Supplement: Supplement 1 [file tvst-10-11-15_s001.pdf]

Supplemental Tables

Supplemental Table 1: Antibodies used in this study. HIER = Heat induced epitope retrieval

Antigen	Host	Dilution	Antigen retrieval	Source
Anti-Iba1 (flat mounts)	Rabbit	1:500	-	ab178846, Abcam
Anti-Iba1 (sections)	Rabbit	1:1000	HIER: pH 9, 95°C, 20 min	019-19741, Wako
Anti-GFAP	Rabbit	1:8000	HIER: pH 9, 95°C, 20 min	Ab7260, Abcam
Anti-CD31	Goat	1:200	Leica Enzyme kit, 37°C, 5 min	AF3628, RnD systems
Anti-GFP	Rabbit	1:1000	Leica Enzyme kit, 37°C, 5 min	Ab290, Abcam
Anti-goat-HRP	Rabbit	1:500	-	5160-2504, Biozol
OPAL Secondary antibodies	unknown	Ready-to-use	-	SKU ARH1001EA, Akoya Biosciences
anti-rabbit Alexa Fluor 568	Goat	1:1000	-	A11011, Molecular Probes

Supplemental Methods

AAV-stuffer sequence

TAAAACAAAACAAAACAAAATAAAACAAAAAAAAGGAAGGAAAAAAAAAGAAAAAATTTA
AAAAATTTTAAAAATATAACGAGGGATAAATTTTTGGTGGTGATAGTGTCCCAGTACAAA
AAGGCTGTAAGATAGTCAACCACAGTAGTCACCTATGTCTGTGCCTCCCTTCTTTATTG
GGGACATGTGGGCTGGAACAGCAGATTTCAGCTACATATATGAACAAATCCTTTATTATT
ATTATAATTATTTTTTTGCGTGAAAGTGTTACATATTCTTTCACTTGTATGTACAGAGAGG
TTTTTCTGAATATTTATTTTAAGGGTTAAATCACTTTTGCTTGTGTTTATTACTGCTTGAG
GTTGAGCCTTTTGAGTATTTAAAAAATATATACCAACAGAACTACTCTCCCAAGGAAAAT
ATTGCCACCATTTGTAGTCCACGTAACCTTCAAGTATGTGCTACTTTTTTGTCCCTGTAT
CTAACTCAAATCAGGAACTGTATTTTTTTTAATGATTTGCTTTTGAAACTTGAAGTCTTGA
AAACAGTGTGATGCAATTACTGCTGTTCTAGCCCCCAAAGAGTTTTCTGTGCAAAATCTT
GAGAATCAATCAATAAAGAAAGATGGAAGGAAGGGAGAAATTGGAATGTTTTAACTGCA
GCCCTCAGAACTTTAGTAACAGCACAACAAATTAAAAACAAAAACAACTCATGCCACAGT
ATGTCGTCTTCATGTGTCTTGCAATGAACTGTTTCAGTAGCCAATCCTCTTTCTTAGTAT
ATGAAAGGACAGGGATTTTTGTTCTTGTTGTTCTCGTTGTTGTTTTAAGTTTACTGGGGA
AAGTGCATTTGGCCAAATGAAATGGTAGTCAAGCCTATTGCAACAAAGTTAGGAAGTTT
GTTGTTTGTTTATTATAAACAAAAAGCATGTGAAAGTGCACTTAAGATAGAGTTTTTATTA
ATTACTTACTTATTACCTAGATTTTAAATAGACAATCCAAAGTCTCCCCTTCGTGTTGCCA
TCATCTTGTTGAATCAGCCATTTTATCGAGGCTCGTGATCAGTGTTGCAACATAATGAAA
AAGATGGCTACTGTGCCTTGTGTTACTTAATCATACAGTAAGCTGACCTGGAAATGAAT
GAAACTATTACTCCTAAGAATTACATTGTATAGCCCCACAGATTAAATTTAATTTATTAAT
TCAAAACATGTTAAACGTTACTTTCATGTACTATGGAAAAGTACAAGTAGGTTTACATTA
CTGATTTCCAGAAGTAAGTAGTTTCCCCTTTCCTAGTCTTCTGTGTATGTGATGTTGTTA
ATTTCTTTTATTGCATTATAAAATAAAAGGATTATGTATTTTTAACTAAGGTGAGACATTG
ATATATCCTTTTGCTACAAGCTATAGCTAATGTGCTGAGCTTGTGCCTTGGTGATTGATT
GATTGATTGACTGATTGTTTTAACTGATTACTGTAGATCAACCTGATGATTTGTTTGTTTG
AAATTGGCAGGAAAAATGCAGCTTTCAAATCATTGGGGGGAGAAAAAGGATGTCTTTCA
GGATTATTTTTATTAATTTTTTTCATAATTGAGACAGAACTGTTTGTTATGTACCATAATG
CTAAATAAAACTGTGGCACTTTTCACCATAATTTAATTTAGTGGAAAAAGAAGACAATGC
TTTCCATATTGTGATAAGGTAACATGGGGTTTTTCTGGGCCAGCCTTTAGAACACTGTTA
GGGTAC

Porcine retinal explants

The eyes of domestic pigs were obtained from the local abattoir (Biberach, Germany) directly *post mortem*, and were transported on ice. Each eye was cleaned from the surrounding tissues in PBS Antibiotic-Antimyotic (Gibco). The eyes were opened with scissors, and, after removal of the cornea, lens and vitreous body, the eye cup was washed in medium, consisting of Neurobasal®-A medium supplemented with 2mM L-glutamine, B-27®supplement, Antibiotic-Antimyotic (all from Gibco). The eye cup was opened with four cuts. The retina was moistened with medium and retinal pieces were excised with a biopsy punch (Ø = 4 mm, Electron Microscopy Sciences). The explants were transferred to Millicell® PCF 6 well membranes (Millipore), ensuring that the photoreceptor layer faced the supporting membrane. The inserts were then placed in a 6-well plate (Millipore) with 1.5ml medium per well, and maintained in an incubator (37°C, 5% CO_2). 50% of the culture medium was replaced every second day. 4 µL of AAV suspension ($1x10^9$ VG/µL) was applied at the first day of culture to the ganglion cell layer side of the tissue.

pVEGFR2 alphaLISA

To measure VEGF activity, porcine retinal explant supernatants were used to stimulate primary Human Retinal Microvascular Endothelial Cells (HRMEC, ACBRI 181, Cell Systems). Phosphorylation of VEGF Receptor 2 (VEGFR2) as a readout for VEGF activity was measured by the AlphaLISA SureFire Ultra™ pVEGFR2 (Tyr1175) assay (PerkinElmer) according to the manufacturer's manual. Samples were measured with an EnVision 2104 Multilabel plate reader (PerkinElmer). Recombinant human VEGF (rhVEGF, 293-VE, RnD systems) was used as positive control.

HEK Blue reporter assays

HEK Blue^{TM} TNF-α and HEK Blue^{TM} IL-6 reporter cells (InvivoGen) stably express alkaline phosphatase under the control of TNF-α and IL-6 inducible promoters, respectively. Porcine retinal explants were used to stimulate HEK Blue reporter cells to test for TNF-α and IL-6 activity. Recombinant human TNF-α (rhTNF-α, 210-TA, RnD Systems) and recombinant human IL-6 (rhIL-6, 206-IL, RnD Systems) were used as positive and negative controls. Alkaline phosphatase activity was measured with the QUANTI-Blue kit (InvivoGen) and a SpectraMax Plus 384 plate reader (Molecular Devices).

Supplemental Figures

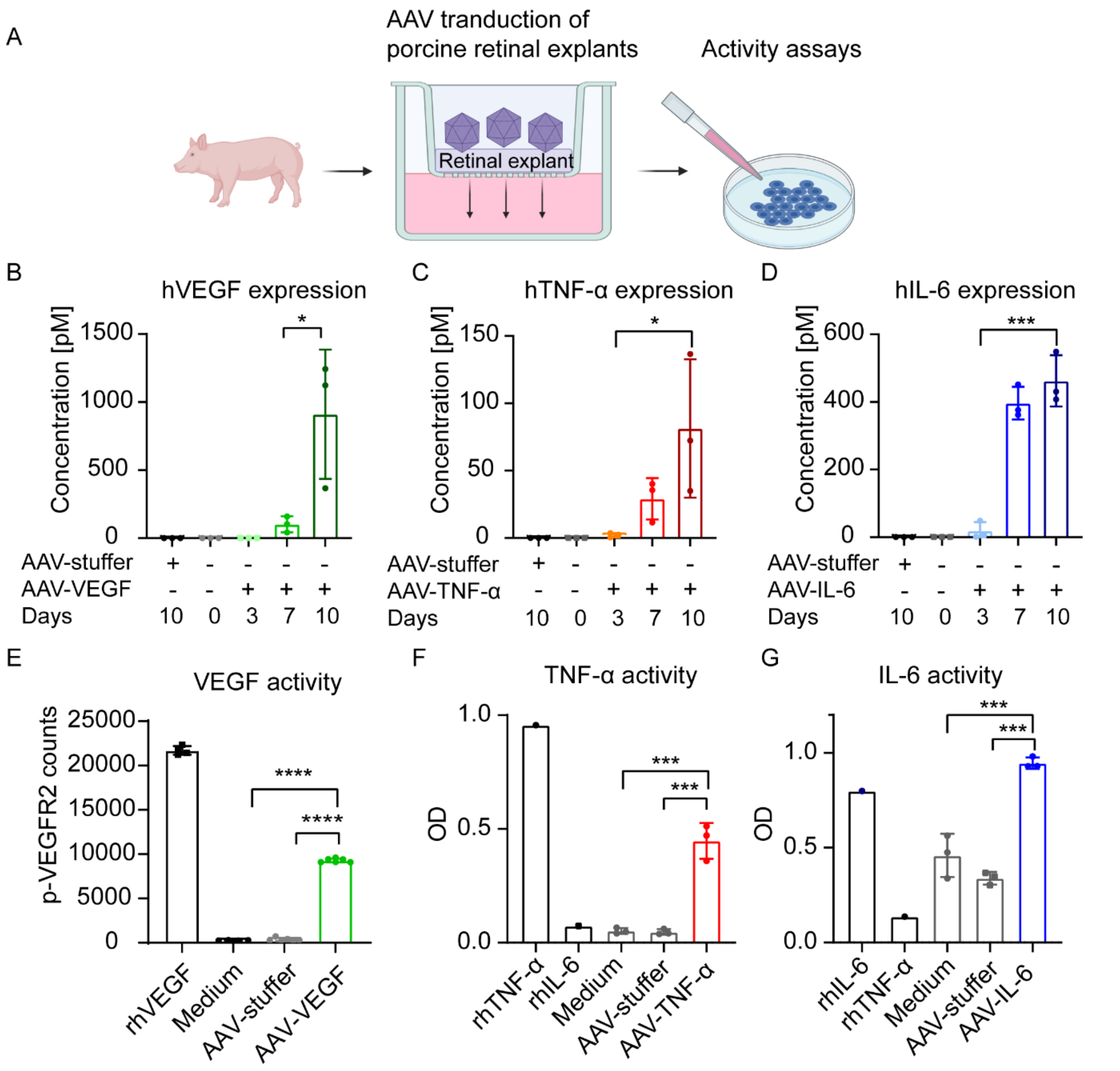

Supplemental Figure 1: Human VEGF, TNF-α and IL-6 were expressed by AAVs in porcine retinal explants and are functionally active. A) Scheme of the experimental setup. Porcine retinal explants are transduced by AAVs and the supernatant has been used to measure the activity of AAV-expressed human VEGF, TNF-α and IL-6. B-D) VEGF (B), TNF-α (C) and IL-6 (D) were expressed in porcine retinal explants 7-10 days after AAV transduction as measured by ELISA (n=3). E) AAV-expressed VEGF in porcine retinal explant supernatants phosphorylated pVEGFR2 in HRMEC cells (****p<0.0001, n=6). F) AAV-expressed TNF-α in porcine retinal explant supernatants activated the TNF-α reporter gene in HEK Blue TNF-α cells (***p<0.001, n=3). G) AAV-expressed IL-6 in porcine retinal explant supernatants activated the IL-6 reporter gene in HEK Blue IL-6 cells (***p<0.001, n=3). Statistical analysis was done by 1-way ANOVA with Tukey's post hoc test.

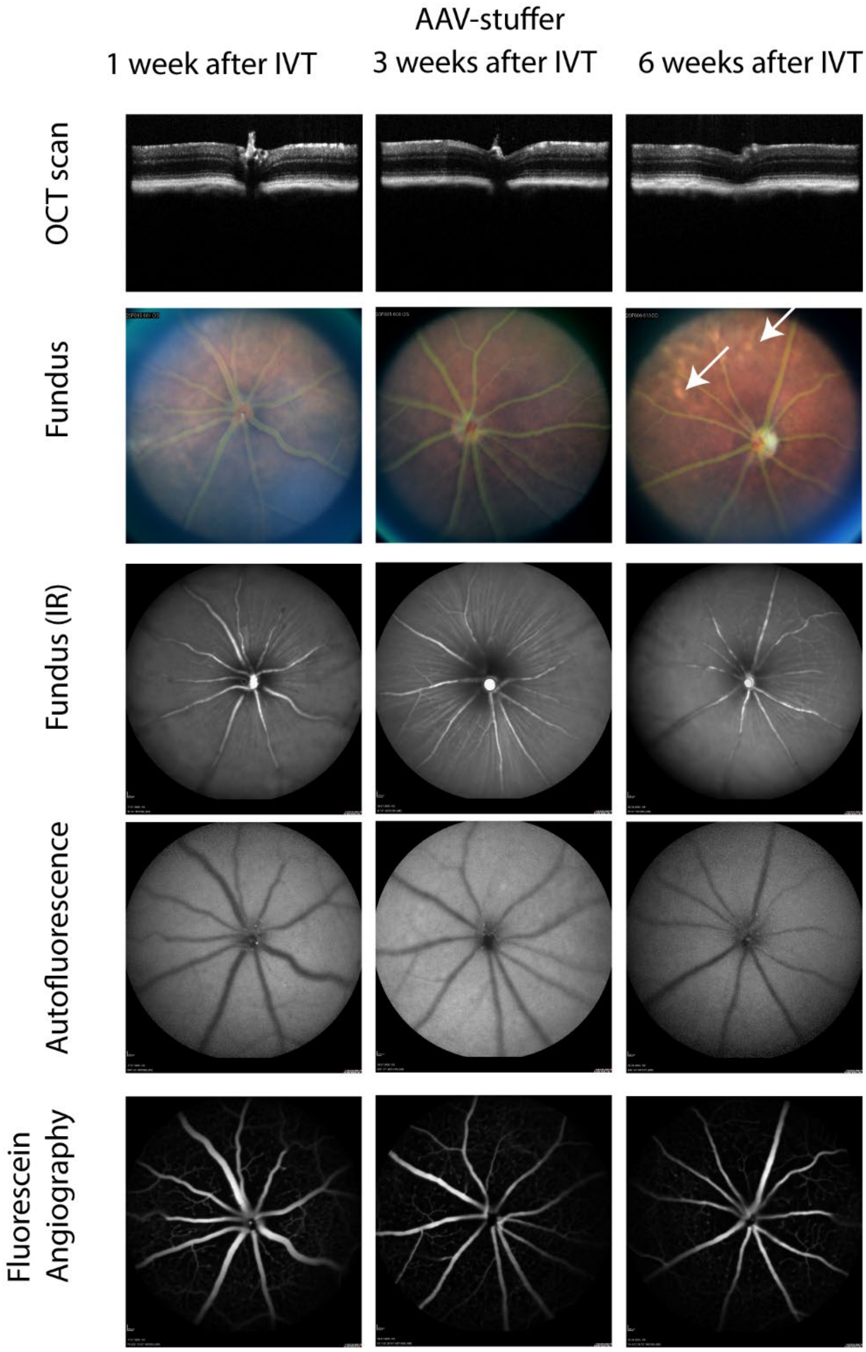

Supplemental Figure 2: *In vivo* imaging anaylsis of AAV-stuffer injected control mice did not reveal any obvious pathologies. OCT scans (top row), fundus pictures, autofluorescence and FFA appeared normal 1-6 weeks after $4x10^9$ VG/eye AAV-stuffer injection In few mice we observed bright unspecific spots in the fundus pictures (white arrow) that were also seen in eyes injected with other ShH10 viruses (see Figure 2A).

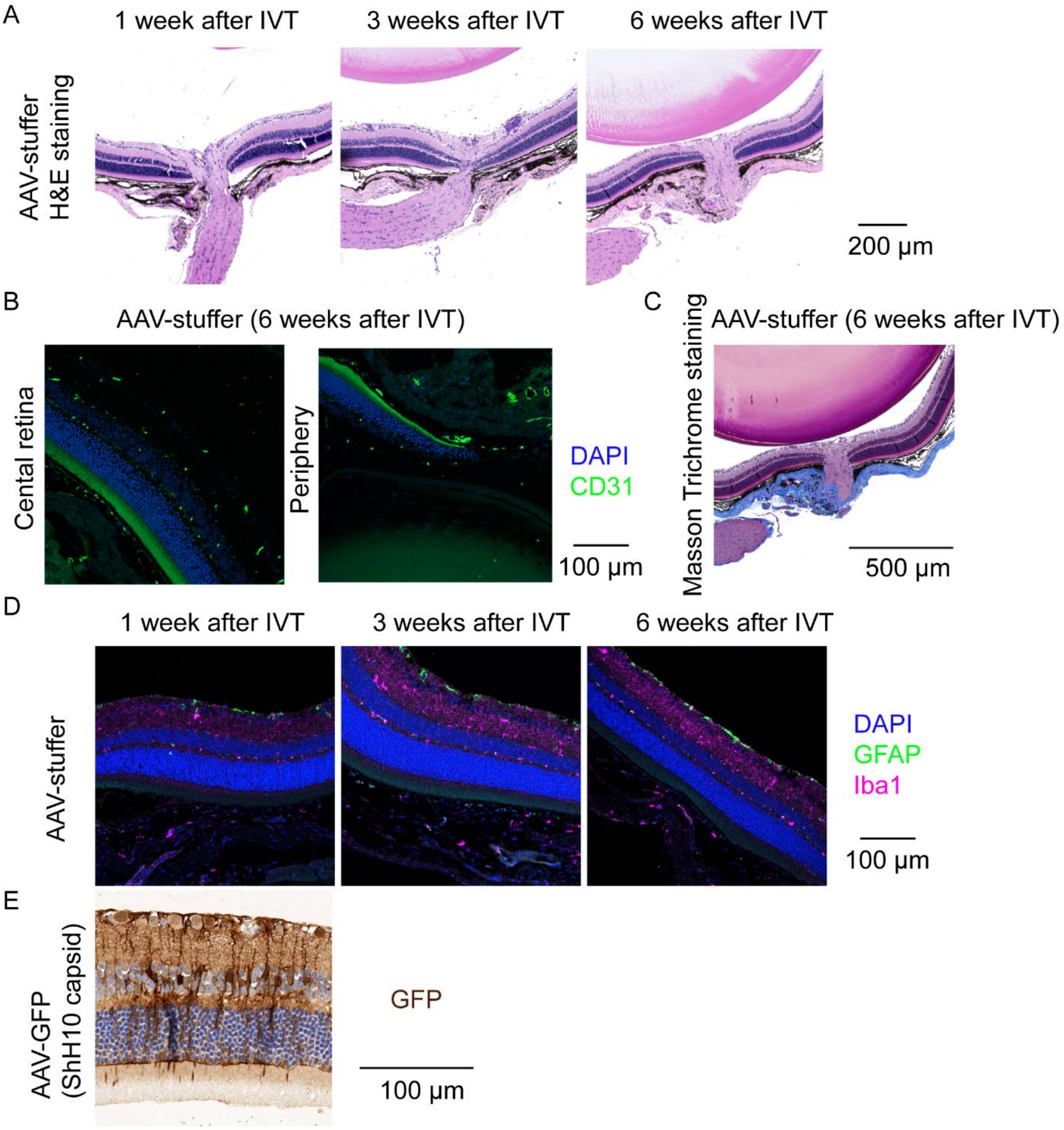

Supplemental Figure 3: Analysis of histological cross-sections of AAV-stuffer injected eyes. A) No major pathologies were observed in H&E stainings of eyes injected with AAV-stuffer ($4x10^9$ VG/eye). B) CD31 immunostaining demonstrated normal vasculature in AAV-stuffer injected eyes (CD31: green, DAPI: blue). C) No collagen (light blue) was found within the retina of AAV-stuffer injected eyes 6 weeks after IVT injection (Masson Trichrome staining). D) Neither $Iba1^+$ microglia/macrophages nor Müller glia (green) were activated in AAV-stuffer injected eyes (Iba1: pink, GFAP: green, DAPI: blue). E) Many Müller glia, but also few RGCs and photoreceptor cells were GFP-positive after IVT injection of $3x10^9$ VG/eye AAV-GFP with the ShH10-capsid (brown: GFP, blue: nuclei).
